# Osteopontin Gene Polymorphism and Urinary OPN Excretion in Patients with Immunoglobulin A Nephropathy

**DOI:** 10.3390/cells8060524

**Published:** 2019-05-31

**Authors:** Beata Kaleta, Natalia Krata, Radosław Zagożdżon, Krzysztof Mucha

**Affiliations:** 1 Department of Clinical Immunology, Medical University of Warsaw, Nowogrodzka 59, 02-006 Warsaw, Poland; radoslaw.zagozdzon@wum.edu.pl; 2 Department of Immunology, Transplantology and Internal Diseases, Medical University of Warsaw, Nowogrodzka 59, 02-006 Warsaw, Poland; natalia.krata@onet.eu (N.K.); kjmucha@gmail.com (K.M.)

**Keywords:** osteopontin, gene, polymorphism, *SPP1*, immunoglobulin A nephropathy, kidney

## Abstract

Osteopontin (OPN) is a glycoprotein involved in the pathogenesis of multiple autoimmune and inflammatory conditions. However, the association of variants of secreted phosphoprotein 1 gene (*SPP1*), which encodes OPN, with immunoglobulin A nephropathy (IgAN) has not been examined up to date. Moreover, the role of OPN in disease pathogenesis and clinical manifestations is not fully known. Therefore, the aim of the study was to determine the frequency of four single nucleotide polymorphisms (SNiPs) of *SPP1* gene, as well as the urinary OPN excretion in IgAN patients and healthy controls. In total, 58 Caucasian patients with biopsy-proven IgAN and 184 gender-, age-, and ethnically-matched healthy controls were genotyped for rs1126616, rs1126772, rs9138, and rs7687316/rs3841116 polymorphisms by real time polymerase chain reaction (RT-PCR). Urinary OPN concentration was determined by enzyme-linked immunosorbent assay (ELISA) in 58 IgAN patients and 19 controls. *SPP1* SNiPs, as well as urinary OPN excretion, were analyzed in relation to their possible associations with the clinicopathological parameters. The frequency of the minor TT/CT genotypes of rs1126616 was significantly higher in IgAN patients compared to controls (P = 0.0217). Similarly, the minor (CC/AC) genotypes and the C allele of rs9138 were more frequent in IgAN patients (P = 0.0425 and P = 0.0112, respectively). Moreover, these two SNiPs were associated with the higher urinary OPN excretion (P < 0.05). These findings suggest that rs1126616, as well as rs9138, may be associated with IgAN development, however future studies in this field are required.

## 1. Introduction

Immunoglobulin A nephropathy (IgAN), also known as Berger disease or synpharyngitic glomerulonephritis, is one of the most common primary glomerulonephritis worldwide [1]. The disease is variable in both its clinical and histopathological presentations. IgAN is characterized by the deposition of IgA (especially galactose-deficient IgA1 (Gd-IgA1)) in the mesangial area of the glomeruli and increased levels of anti-Gd-IgA1 autoantibodies [2]. Moreover, co-deposits of IgG, IgM, and complement C3 are also observed [3]. These immune complexes induce the proliferation of mesangial cells and increase the production of extracellular matrix proteins, as well as cytokines and chemokines [3,4]. IgAN is usually asymptomatic in the early stage. In patients, microscopic and macroscopic hematuria, nephrotic syndrome, and acute reversible renal failure can be observed [5]. The disease occurs more often in men (the ratio of male to female is 6:1 in Europe) [6]. Moreover, recent studies demonstrated that renal progression occurs faster in males than in females, however some reports suggested no gender differences [7,8]. Although genetic and environmental factors appear to play a role in the pathogenesis of IgAN, the etiology of the disease remains to be elucidated.

Osteopontin (OPN) is a pleiotropic glycoprotein, expressed in bone, immune cells, smooth muscle, epithelial and endothelial cells, neurons, adipocytes, Kupffer cells, and many others. Moreover, OPN is present in kidneys and secreted into urine [9,10]. This protein is involved in the bone remodeling, but also acts as a regulator of immune response. It was demonstrated that OPN regulates migration and infiltration of macrophages, T lymphocytes, and dendritic cells [11]. Moreover, OPN enhances antibody production, regulates nitric oxide synthesis, and stimulates interleukin (IL)-17 production [10,11]. Recent studies showed that OPN plays a key role in the regulation of apoptosis, angiogenesis, and in cancer progression [9,10]. OPN interacts with multiple integrins and CD44v3 receptor, and thus modulates the phosphorylation of kinases, which are involved in the nuclear factor kappa B (NFκB) activation and cytokine production [12]. Moreover, OPN regulates T cell chemotaxis, adhesion, and IL-10 production by macrophages [12,13,14]. It was revealed that the expression and biological activity of OPN can be modulated by polymorphism of its gene [15].

Numerous studies demonstrated that OPN encoded by secreted phosphoprotein 1 (SPP1) gene, as well as polymorphism of the *SPP1* gene, are associated with the pathogenesis and progression of some autoimmune diseases [16,17,18,19,20,21,22,23]. Moreover, recent investigations provided new insights into the role of OPN in the pathogenesis of various kidney diseases (reviewed in a previous study [24]).

Although some reports suggested that OPN participates in kidney failure, the association of OPN and its gene polymorphisms with IgAN is not fully known.

Therefore, the aim of the study was to determine the frequency of four single nucleotide polymorphisms (SNiPs) of *SPP1* gene—rs1126616, rs1126772, rs9138, rs7687316/rs3841116—and urinary OPN excretion in IgAN patients and healthy controls, and their possible associations with the clinicopathological parameters.

## 2. Materials and Methods

### 2.1. Patients and Sample Collection

The study was approved by the Ethics Committee of Medical University of Warsaw (No. KB/9/2010) and all subjects provided written informed consent. The procedures followed were in accordance with the Helsinki Declaration of 1975, as revised in 2000.

Urine samples (second morning urine) were collected from 58 Caucasian patients with IgAN, who visited the Department of Immunology, Transplantology, and Internal Diseases, Medical University of Warsaw. IgAN was defined by a renal biopsy, demonstrating dominant IgA deposits in the mesangium of glomeruli by immunofluorescence microscopy. Patients with evidence of secondary IgAN and other systemic or autoimmune diseases were excluded. The age-, gender-, and ethnically-matched 19 healthy controls were randomly selected after health examinations in the same hospital. Urine samples were centrifuged (10 min at 2000 RPM) within 30 min of collection and frozen at the temperature of −80 °C.

Venous blood samples were collected in the tubes containing ethylene diaminetetraacetic acid (EDTA) from 58 IgAN patients and 184 controls. The control group consisted of healthy volunteers, unrelated with IgAN patients, with no family history of any kidney diseases. IgAN patients were age-, gender-, and ethnically-matched with controls at 1:2 or 1:3 ratio. The short characteristics of studied groups are shown in Table 1.

### 2.2. Selection of SPP1 Polymorphisms

The PubMed database was used to find relevant literature and identify OPN as a protein which had a confirmed relevant role in the immunomodulation and possible role in kidney biology, according to the published studies. Selection of polymorphisms in the *SPP1* gene for genotyping in this study was done by using the HapMap database (http://www.hapmap.org/). Three of four selected polymorphisms—rs1126616 (in exon 7), rs1126772, and rs9138 (in 3′-untranslated region)—have a minor allele frequency of >5% in Caucasians. Additionally, rs7687316/rs3841116 (in the promoter), together with rs1126616, rs1126772, and rs9138, was previously associated with some autoimmune diseases [17]. This information prompted us to study the possible association between these four *SPP1* polymorphisms and IgAN.

### 2.3. Genotyping of the SPP1 Gene rs1126616, rs1126772, rs9138, rs7687316/rs3841116 Polymorphisms

Genomic DNA was extracted from whole frozen blood using “Blood Mini” kit (A&A Biotechnology, Poland) according to the manufacturer’s instructions. DNA concentration and purity were determined with UV spectrophotometry, measuring absorbance ratios of 260/280 nm. All genomic DNA was diluted to a final concentration of 20 ng/µL.

*SPP1* rs1126616, rs1126772, rs9138, and rs7687316/rs3841116 genotypes were analyzed using a real-time PCR method. Genotyping was carried out with the LightSNiP typing assay (TIB-MolBiol, Germany) by analyzing the melting curves with the LightCycler^®^ 480 system available from Roche Diagnostics. Real-time PCR reactions were performed in 96-well PCR plates with cycling conditions as optimized by TIB-MolBiol.

### 2.4. Urine OPN Measurement

The concentration of full-length OPN in the urine was measured in duplicates by Human Osteopontin (OPN) ELISA Kit (DL Sci and Tech Development Co, Wuxi, China), according to the manufacturer’s instructions. Chromate 4300 Microplate Reader was used for reading at 450 nm. The results were expressed in ng/mL. The detection limit was 0.234 ng/mL.

### 2.5. Statistical Analysis

Statistical analysis was performed using the R software, version 3.5.3. Allelic and genotypic frequencies were obtained by direct counting. All genotyping results were tested for HWE, evaluated with the χ^2^ test. The allelic and genotypic frequencies were compared between the IgAN patients and controls using the χ^2^ with Yate’s correction test with 2 × 2 and 2 × 3 contingency tables. Differences in OPN urinary levels, *SPP1* genotypes/alleles, and clinicopathological parameters were evaluated by the Mann-Whitney U test, ANOVA, or the Tukey HSD test, as appropriate. Differences were considered statistically significant at a P value < 0.05.

## 3. Results

### 3.1. SPP1 Polymorphisms Frequency

The frequency of the SPP1 rs1126616, rs1126772, rs9138, and rs7687316/rs3841116 genotypes and alleles between the two groups is summarized in the Table 2. The frequency of all analyzed variants were in HWE for IgAN patients and controls (P > 0.05).

As shown in Table 2, the frequency of the homozygous minor TT genotype of SPP1 rs1126616 polymorphism was significantly increased in IgAN patients compared to healthy controls (P = 0.0217). Moreover, the chi-square analysis demonstrated a trend for higher frequency of the T allele in IgAN patients compared to the control group (P = 0.0531). The results suggest that rs1126616 might play a role in the pathogenesis of IgAN. The analysis of frequency of rs9138 genotypes and alleles likewise demonstrated that this polymorphism can be associated with IgAN. The minor CC genotype and the C allele were more prevalent among patients than controls (P = 0.0425 and P = 0.0112, respectively). All the participants were also genotyped for rs1126772. The wild type (major) AA genotype and allele A of this polymorphism were the most prevalent in both groups and the analysis did not show a significant association between these genetic variants and IgAN (P > 0.05). Moreover, the genotyping results for rs7687316/rs3841116 showed no significant differences in the comparison of genotype and allele frequencies between patients and controls (P > 0.05), which suggests that this polymorphism is not associated with the risk of IgAN.

In addition, the analysis of influence of SPP1 rs1126616, rs1126772, rs9138, and rs7687316/rs3481116 variants on clinical parameters demonstrated that these SNiPs are not associated with serum creatinine, glomerular filtration rate (GFR), hemoglobin, and 24-h proteinuria in IgAN patients (P > 0.05, data not shown).

### 3.2. Urinary OPN Levels

**Figure 1:**
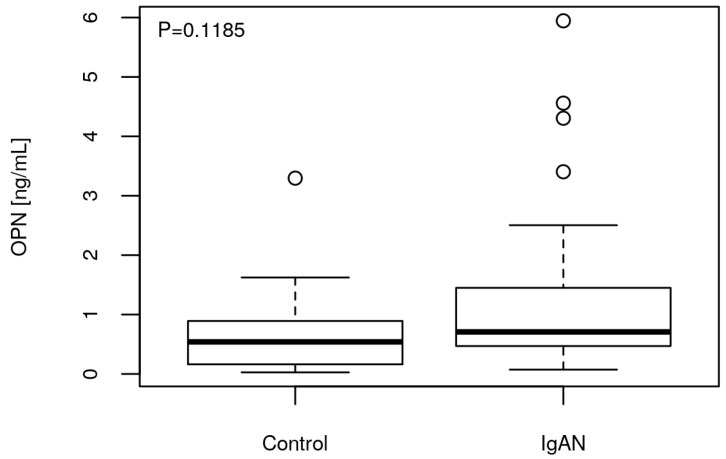
Comparison of median urinary osteopontin (OPN) levels between immunoglobulin A nephropathy (IgAN) patients (N = 58) and controls (N = 19).

The mean urine concentration of OPN in IgAN patients was higher than in healthy controls (1.14 ng/mL vs. 0.72 ng/mL). The trend was positive but statistically insignificant (P = 0.1185) (Figure 1). However, no association of urinary OPN levels with serum creatinine, GFR, hemoglobin, 24-h proteinuria, and age in IgAN patients was found (P > 0.05, data not showed).

Analysis of OPN concentration in urine of IgAN patients in relation to the *SPP1* genotypes demonstrated that OPN excretion was associated with minor rs1126616, rs1126772, and rs9138 genotypes, but not with rs7687316/rs3481116 variants (Table 3).

Mean OPN levels were 0.57 ng/mL in individuals with the CC genotype of rs1126616, 1.23 ng/mL in patients carrying CT genotype and 2.39 ng/mL in those with TT genotype (P = 0.0083 for CC vs. CT, P = 0.0014 for CC vs. TT). These results strongly suggest that minor alleles of rs1126616 may predispose to higher OPN urine excretion in IgAN patients.

The analysis of OPN concentration in relation to rs1126772 genotypes likewise demonstrated that this polymorphism is associated with higher OPN excretion. Mean OPN levels were 0.62 ng/mL in IgAN patients with AA genotype, 1.13 ng/mL in individuals with AG genotype, and 2.52 ng/mL in those with GG genotype (P = 0.0355 for AA vs. AG, P = 0.0041 for AA vs. GG).

Mean OPN concentration in urine of IgAN patients with AA genotype of rs9138 was 0.36 ng/mL, 1.05 ng/mL in individuals with AC genotype, and 2.21 ng/mL in those with CC genotype (P = 0.0060 for AA vs. AC, P = 0.001 for AA vs. CC), which indicates that this SNiP is associated with urinary OPN excretion.

The study demonstrated that rs7687316/rs3481116 polymorphism does not affect the OPN levels. Mean OPN concentration in urine of IgAN patients with TG genotype was 2.59 ng/mL and 1.02 ng/mL in individuals with TT genotype (P > 0.05).

## 4. Discussion

OPN is a glycoprotein involved in multiple physiological and pathological processes [9,11]. Recent studies suggested that OPN could be a promising biomarker for various kidney diseases (revised in a previous study [24]), however the association of OPN and its gene polymorphisms with IgAN is not fully understood.

The present study was conducted to determine the frequency of rs1126616, rs1126772, rs9138, and rs7687316/rs3481116 SNiPs in the *SPP1* gene, as well as urinary OPN excretion in adult patients with IgAN and in healthy controls and their possible association with disease manifestations.

Recent investigations revealed that rs1126616, as well as rs1126772 and rs9138 SNiPs, are associated with higher OPN mRNA stability [25]. In turn, rs7687316/rs3481116 modulates the transcriptional activity of the *SPP1* gene [26].

In the present study we demonstrated for the first time that the minor (TT and CT) genotypes of rs1126616 polymorphism were more frequent in IgAN patients than in healthy controls and were associated with higher urinary OPN excretion. The analysis of rs9138 variants likewise demonstrated that this SNiP can predispose to IgAN. The minor (CC and AC) genotypes and the C allele were more prevalent among IgAN patients than controls and were associated with elevated urinary OPN levels. Moreover, the minor variants of rs1126772 (GG and AG) were also associated with elevated OPN in the urine of IgAN patients. We believe, that these three SNiPs, through their effects on increased *SPP1* gene transcriptional activity [25,26], contribute to the increase of OPN excretion in the urine. One of the *SPP1* SNiPs examined in the present study (rs1126616) was also genotyped by Forton and colleagues [26] in patients with SLE. It was found that the T allele is associated with higher risk of lupus nephritis (LN) development. In another similar study [17] it was revealed that the frequency of CT and TT genotypes of rs1126616 was higher in SLE patients with LN compared to those without LN. These reports suggest that this polymorphism may be a risk factor of renal damage. Another study likewise suggested that 9250C/T polymorphism is associated with susceptibility to LN in Chinese Han SLE patients [27]. The association of rs11730582 polymorphism and pathogenesis of nephropathy in diabetic patients was also examined. It was found that the T allele and TT genotype of this SNiP are associated with the higher risk of DN, higher proteinuria, and lower GFR [28,29]. Cheema and colleagues [28] studied the association of rs11730582, rs17524488, and rs28357094 *SPP1* variants in another case-control study but the group found that only the GG genotype of rs17524488 is associated with the lower risk of DN and higher GFR.

In the present study we also compared the mean urine levels of OPN in IgAN patients and healthy controls. We demonstrated that urine OPN concentration was higher in patients than in the control group, however the positive trend was statistically insignificant. Moreover, no association of urinary OPN levels or *SPP1* gene polymorphisms with serum creatinine, GFR, hemoglobin, or 24-h proteinuria was found. However, we showed that OPN level in urine of IgAN patients is associated with rs1126616, rs1126772, and rs9138 variants. These results suggest that rather than OPN, *SPP1* polymorphism may play a possible role in IgAN.

Sano and colleagues likewise examined whether OPN participates in the IgAN pathogenesis and progression [30]. The group found that OPN and its CD44 receptor were expressed in the tubular cells and interstitial infiltrating cells in areas of tubulointerstitial injury. Moreover, their expression was associated with the proteinuria and creatinine clearance rate, which was in contrast to our study. In another study, Wasilewska et al. [31] demonstrated that the urine concentration of OPN in pediatric IgAN patients is higher than in healthy controls, which was in line with our observations in adult patients. In addition, the group found that urine OPN levels correlated with macrophage infiltration during IgAN progression. Gang et al. [32] likewise measured OPN concentration in urine of IgAN patients and reported that the N-half form of this protein was positively correlated with albuminuria. These reports suggested that OPN could play a possible role in IgAN development, however, some studies gave opposite results. Kitagori and coworkers [33] did not find significant difference in the urine OPN concentration between IgAN patients and healthy controls. Another study showed no correlation of OPN expression and pathological changes in the renal tissue of IgAN patients either [34]. Therefore, future studies in this field are needed to better define and explore the role of OPN in IgAN pathogenesis and progression.

Recent investigations suggested that OPN is associated with other kidney diseases. It was demonstrated that this protein plays an important role in the growth and invasion of human renal cancer [35,36,37] and its elevated expression is associated with the poor progression-free survival in patients with clear cell renal cell carcinoma [37]. The association between OPN and minimal change disease (MCD) and focal and segmental glomerulosclerosis (FSGS) was also investigated and it was found that urinary OPN/creatinine ratio in both groups was significantly higher than in healthy controls and was associated with interstitial changes and mesangial expansion [31]. The work of Gang et al. [32] revealed that in MCD patients OPN mRNA expression correlates with the urinary OPN excretion, however, similar study conducted by Kitagori and colleagues showed no difference in OPN levels between MCD patients and healthy controls [33]. In another study higher expression of OPN in kidneys of MCD patients compared to controls was demonstrated [38]. Similar findings were observed in patients with membranous glomerulonephritis (MGN), in which OPN correlated with increased infiltration of macrophages, as well as CD4^+^ and CD8^+^ T cells [38,39]. The role of OPN in the pathogenesis of diabetic nephropathy (DN) was also examined. It was found that OPN concentration in plasma, but not in urine of patients with DN, was higher than in controls, especially during the disease progression [40,41]. Moreover, a negative correlation between OPN and GFR was found. Elevated plasma OPN level was also observed in pediatric and adult patients with type 1 diabetes (T1D), in which OPN was associated with the development of microalbuminuria and was a strong predictor of incipient DN [42,43]. A large number of studies demonstrated that OPN level is increased in plasma and urine of patients with SLE, especially in patients with LN [17,44,45], but some studies gave opposite results. Kitagori and colleagues [33] showed no difference in urine OPN excretion between LN patients and controls, but plasma (full-length and N-half) OPN level was higher in LN group. Moreover, both plasma and urine OPN were not associated with SLE Disease Activity Index (SLEDAI) and GFR. In another study Ma et al. revealed that higher OPN expression in LN patients was associated with intrarenal macrophage infiltration and podocyte injury [46].

## 5. Conclusions

The present study strongly suggests an association between *SPP1* gene rs1126616 and rs9138 polymorphisms and IgAN susceptibility, however, the results need to be replicated on a larger sample. An additional limitation of the present study is that plasma OPN concentration was not measured, which could add a stronger support to the final conclusions and could be correlated with the *SPP1* genotype-dependent differences.

The role of *SPP1* variants in kidney diseases needs to be better explored as a diagnostic target to monitor these conditions. Advances in understanding specific *SPP1* SNiPs may be helpful to create genetic profiles for predisposition to autoimmune diseases, including IgAN.

## Figures and Tables

**Table 1:** Characteristics of IgAN patients and controls. Urine samples were collected for OPN concentration measurement. Blood samples were collected for genotyping of *SPP1* gene polymorphisms.

Parameter	Urine Samples		Blood Samples	
	IgAN Patients N = 58	Controls N = 19	IgAN Patients N = 58	Controls N = 184
Age, year	43.84 ± 14.46	48.89 ± 13.77	43.84 ± 14.46	42.6 ± 12.8
Gender (male/female), n	33/25	9/10	33/25	99/85
Creatinine (mg/mL)	1.27 ± 0.52	0.87 ± 0.14	-	-
GFR (mL/min/1.73 m^2^)	73.32 ± 29.37	91.03 ± 12.26	-	-
Hemoglobin (g/L)	14.43 ± 1.61	14.04 ± 1.21	-	-
Total protein (24-h excretion, mg/d)	0.64 ± 0.13	-		

Note: IgAN, immunoglobulin A nephropathy; GFR, glomerular filtration rate.

**Table 2:** Genotypes and alleles frequency of *SPP1* rs1126616, rs1126772, rs9138, and rs7687316/rs3481116 polymorphisms in IgAN patients and controls. Data are expressed as N (%).

	Genotypes			Alleles	
rs1126616					
	CC	CT	TT	C	T
IgAN patients (%)	31 (53)	19 (33)	8 (14)	81 (70)	35 (30)
Control (%)	114 (62)	63 (34)	7 (4)	291 (79)	77 (21)
Association	χ^2^ = 7.66, df = 2, P = 0.0217			χ^2^ = 3.74, df = 1, P = 0.0531	
rs1126772					
	AA	AG	GG	A	G
IgAN patients (%)	32 (55)	19 (33)	7 (12)	83 (72)	33 (28)
Control (%)	105 (57)	64 (35)	15 (8)	274 (74)	94 (26)
Association	n.s.			n.s.	
rs9138					
	AA	AC	CC	A	C
IgAN patients (%)	22 (38)	25 (43)	11 (19)	69 (59)	47 (41)
Control (%)	101 (55)	65 (35)	18 (10)	267 (73)	101 (27)
Association	χ^2^ = 6.32, df = 2, P = 0.0425			χ^2^ = 6.50, df = 1, P = 0.0112	
rs7687316/rs3481116					
	TT	TG	GG	T	G
IgAN patients (%)	56 (97)	2 (3)	0 (0)	114 (98)	2 (2)
Control (%)	183 (99)	1 (1)	0 (0)	367 (99)	1 (1)
Association	n.s.			n.s	

Note: IgAN, immunoglobulin A nephropathy; OPN, osteopontin; n.s. not statistically significant.

**Table 3:** Association of *SPP1* polymorphisms and urinary OPN excretion in IgAN patients.

*SPP1* Genotypes	Mean Urinary OPN Concentration (ng/mL)	P-Value
rs1126616		
CC	0.57	
CT	1.23	0.0083 (CT vs. CC)
TT	2.39	0.0014 (TT vs. CC)
rs1126772		
AA	0.62	
AG	1.13	0.0355 (AG vs. AA)
GG	2.52	0.0041 (GG vs. AA)
rs9138		
AA	0.36	
AC	1.05	0.0060 (AC vs. AA)
CC	2.21	0.0001 (CC vs. AA)
rs7687316/rs3481116		
TC	2.59	
TT	1.02	n.s.

Note: IgAN, immunoglobulin A nephropathy; OPN, osteopontin; n.s. not statistically significant.
